# Toxicity of pathogenic ataxin-2 in *Drosophila* shows dependence on a pure CAG repeat sequence

**DOI:** 10.1093/hmg/ddab148

**Published:** 2021-06-02

**Authors:** Leeanne McGurk, Olivia M Rifai, Oksana Shcherbakova, Alexandra E Perlegos, China N Byrns, Faith R Carranza, Henry W Zhou, Hyung-Jun Kim, Yongqing Zhu, Nancy M Bonini

**Affiliations:** Division of Cell & Developmental Biology, School of Life Sciences, University of Dundee, Dundee, UK; Department of Biology, University of Pennsylvania, Philadelphia, PA, USA; Department of Biology, University of Pennsylvania, Philadelphia, PA, USA; Department of Biology, University of Pennsylvania, Philadelphia, PA, USA; Neurosciences Graduate Group, University of Pennsylvania, Philadelphia, PA, USA; Neurosciences Graduate Group, University of Pennsylvania, Philadelphia, PA, USA; Medical Sciences Training Program, Perelman School of Medicine, University of Pennsylvania, Philadelphia, PA, USA; Department of Biology, University of Pennsylvania, Philadelphia, PA, USA; Department of Biology, University of Pennsylvania, Philadelphia, PA, USA; Department of Biology, University of Pennsylvania, Philadelphia, PA, USA; Department of Biology, University of Pennsylvania, Philadelphia, PA, USA; Department of Biology, University of Pennsylvania, Philadelphia, PA, USA; Neurosciences Graduate Group, University of Pennsylvania, Philadelphia, PA, USA

## Abstract

Spinocerebellar ataxia type 2 is a polyglutamine (polyQ) disease associated with an expanded polyQ domain within the protein product of the *ATXN2* gene. Interestingly, polyQ repeat expansions in *ATXN2* are also associated with amyotrophic lateral sclerosis (ALS) and parkinsonism depending upon the length of the polyQ repeat expansion. The sequence encoding the polyQ repeat also varies with disease presentation: a pure CAG repeat is associated with SCA2, whereas the CAG repeat in ALS and parkinsonism is typically interrupted with the glutamine encoding CAA codon. Here, we asked if the purity of the CAG sequence encoding the polyQ repeat in *ATXN2* could impact the toxicity of the ataxin-2 protein *in vivo* in *Drosophila*. We found that ataxin-2 encoded by a pure CAG repeat conferred toxicity in the retina and nervous system, whereas ataxin-2 encoded by a CAA-interrupted repeat or CAA-only repeat failed to confer toxicity, despite expression of the protein at similar levels. Furthermore, the CAG-encoded ataxin-2 protein aggregated in the fly eye, while ataxin-2 encoded by either a CAA/G or CAA repeat remained diffuse. The toxicity of the CAG-encoded ataxin-2 protein was also sensitive to the translation factor eIF4H, a known modifier of the toxic GGGGCC repeat in flies. These data indicate that ataxin-2 encoded by a pure CAG versus interrupted CAA/G polyQ repeat domain is associated with differential toxicity, indicating that mechanisms associated with the purity of the sequence of the polyQ domain contribute to disease.

## Graphical Abstract

**Graphical Abstract:**
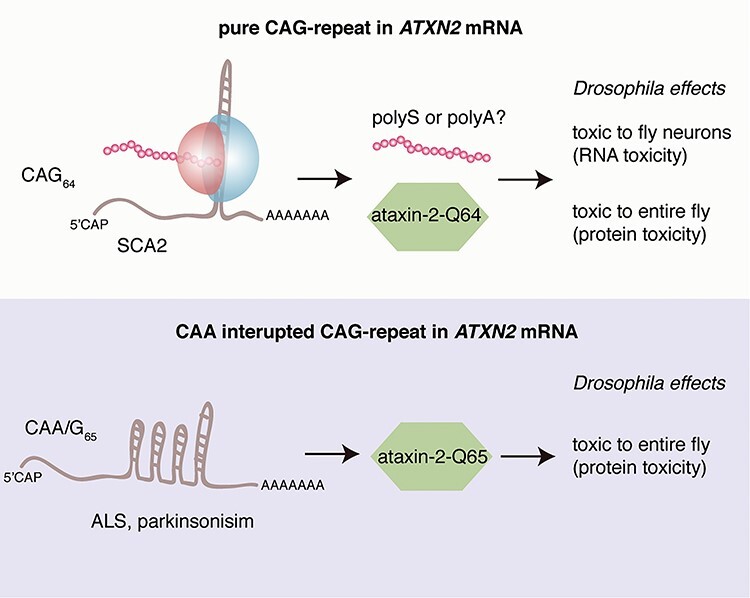

## Introduction

Expansions of microsatellite repeats are a cause of several neurodegenerative disorders. A notable example is the polyglutamine (polyQ) diseases, which are caused by an expansion of a glutamine-encoding CAG-repeat in the respective disease genes, and includes six spinocerebellar ataxias (SCA1, 2, 3, 6, 7 and 17), Huntington’s disease and dentatorubral pallidoluysian atrophy (1,2). Despite the CAG-repeat mutations occurring in a diverse set of proteins, the polyQ diseases share some key pathological mechanisms. For example, longer CAG-repeat expansions result in earlier disease onset and more severe symptoms (2). Furthermore, an expanded polyQ results in aggregation of the disease protein, which causes toxicity via gain-of-function and loss-of-function effects (1,2). An additional mechanism shared by many of the diseases caused by expansions of microsatellites (e.g. CAG, CTG and GGGGCC expansions) is toxicity induced by the structure of the expanded RNA (3–5). RNA toxicity, first implicated in myotonic dystrophy type 1, can lead to effects by binding to and sequestering key cellular RNA-binding proteins such as splicing factors (6–8). RNA with expanded repeats can also bind to translation factors and in doing so, the RNA primes repeat-associated non-AUG protein translation (RAN), which can occur in multiple reading frames and generates peptides (e.g. poly-glutamine, poly-serine and poly-alanine) that accumulate in the brain and are toxic to cells (9–15). Although it is known that the CAG-repeat from SCA2, SCA3 and Huntington’s disease can give rise to toxicity when the repeat is either isolated or flanked by short regions of coding sequence (12,16,17), less is known about the RNA-toxicity that arises from the CAG-repeat when embedded in the entire transcript.

The gene encoding the ataxin-2 protein (*ATXN2*) harbors a CAG-repeat that normally consists of 22 or 23 repeats interrupted with two glutamine-encoding CAA codons (18–20). Expansion of the CAG-repeat in *ATXN2* to >33 causes SCA2, an adult-onset ataxia that primarily affects neurons in the cerebellum and brainstem (2,21). The disease-causing repeat in SCA2 is a pure CAG-tract and lacks the CAA interruption observed in the normal allele (18–20). Protein toxicity is thought to occur in SCA2 as evidenced by the aggregation of polyQ-expanded ataxin-2 protein in the cytoplasm of affected neurons (22,23). The ataxin-2 protein functions in translation and RNA metabolism (2,24,25). A key RNA-binding protein modulated by ataxin-2 is TDP-43 (26). TDP-43 is central to the motor neuron disease amyotrophic lateral sclerosis (ALS) and is mislocalized to the cytoplasm of motor neurons in >95% of patients with ALS (27). In animal and cellular models, ataxin-2 is a dose-sensitive modifier of TDP-43 whereby upregulation of ataxin-2 promotes TDP-43 neurotoxicity and downregulation of ataxin-2 mitigates TDP-43-induced toxicity (26,28,29). Thus, the function of ataxin-2 appears crucial to additional disease contexts.

Clinical, pathological and genetic evidence suggests that SCA2 may be on a disease spectrum with parkinsonism and ALS. For example, some patients harboring an SCA2 expansion can present with parkinsonism or motor neuron disease (30–35). Cytoplasmic accumulation of the ALS protein TDP-43 has been observed in SCA2 brain tissue (26), suggesting that the degeneration in SCA2 and ALS may impact similar cellular pathways. Furthermore, intermediate polyQ expansions of ~32–35Q are associated with parkinsonism (36) and ~29–33Q are associated with ALS (26,37,38). Intriguingly, and in contrast to the SCA2 polyQ expansion, the intermediate polyQ expansion associated with ALS and parkinsonism is often interrupted with CAA codons (39,40). Why the subtle differences in CAG/polyQ-repeat length and composition can lead to different disease presentations is unclear. Elucidating the disease mechanisms that underlie the different CAG repeats will be key for understanding the biological features that contribute to differences in neuronal vulnerability and disease presentation.

**Figure 1:**
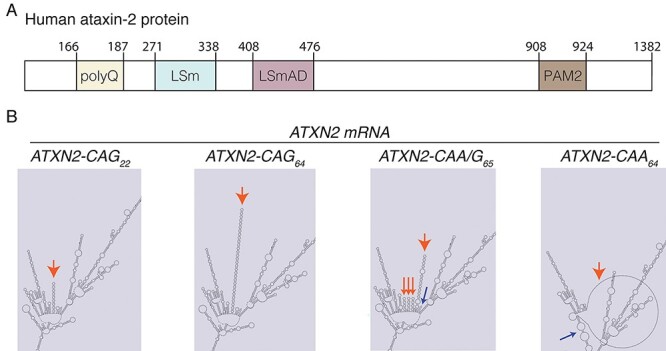
Ataxin-2 with different RNA sequences of the polyQ repeat region. (**A**) Domain structure of ataxin-2 protein. PolyQ: polyglutamine domain, LSm: like-Sm domain, LSmAD: LSm associated domain and PAM2: PolyA-binding protein interacting motif. (**B**) Predicted RNAfold structure of the ataxin-2 RNA with different CAG purity polyQ repeat sequences. The red arrows indicate the CAG, CAA/G or CAA tract in the mRNA sequence, blue arrows indicate regions outside of the repeat that are altered by the CAA/G or CAA expansion.

To better understand how the composition of the CAG-repeats impact *ATXN2*-associated toxicity, we developed *Drosophila* that were transgenic for the human ataxin-2 protein encoded by either a pure CAG repeat, a CAG-repeat interrupted with CAA (CAA/G) or by the extreme non-CAG repeat of a pure CAA sequence. Surprisingly, our data indicate that ataxin-2 expressed from a pure CAG repeat confers toxicity in ways that ataxin-2 expressed from a CAA/G interrupted repeat or pure CAA repeat does not, suggesting that RNA toxicity is a component of the ataxin-2 CAG-repeat expansion. Our novel ataxin-2 fly model presents a highly manipulable genetic system to dissect toxic mechanisms associated with a pure CAG repeat in the context of the ataxin-2 protein.

## Results

### Transgenic constructs of ataxin-2 with different polyQ DNA sequences

To explore the possibility that alternative disease-causing mechanisms are associated with different types of repeats encoding polyQ, we generated a series of constructs and transgenic *Drosophila* that encoded human *ATXN2* with either a pure CAG repeat, an interrupted CAA/G repeat or a pure CAA repeat (Fig. 1). Our overall approach was to assess the toxicity and degeneration that arises from the different ataxin-2 proteins when they are selectively expressed in the *Drosophila* eye. Our previous data indicated that shorter repeat expansions in *ATXN2* (CAG_22_ or CAG_32_) confer little or no visible effects on the eye (28). In human disease, the CAG-repeat length in SCA2 is in the range of 35–59, but it can be as long as 77 repeats (18–20,41). Given that CAG-repeat length in *ATXN2* negatively correlates with disease severity, we assessed the effect of a CAG-repeat of 64 units in the context of the human ataxin-2 protein.

We designed constructs encoding human ataxin-2 with either a pure-CAG repeat of 64 units in length, a CAG repeat interrupted with CAA (CAA/G) in a pattern seen in ALS patients (40), or with the extreme of a pure CAA repeat. Structurally, RNAs with a pure CAG repeat fold into a hairpin, whereas CAA-interrupted repeats break the hairpin and take on a different structure (42). To gain insight into the structures that the different CAG repeat regions (CAG, CAA/G and CAA) are predicted to take in the context of the *ATXN2* mRNA, we used the RNAfold webserver (43). This showed that the expanded CAG, CAA/G and CAA repeat sequences in the *ATXN2* mRNA are predicted to undertake very different structures (Fig. 1B): as expected, the pure CAG is a predicted hairpin, whereas the CAA/G repeat is a series of smaller hairpins, while the CAA forms a large loop (Fig. 1B). Thus, within the context of the entire *ATXN2* mRNA sequence, the CAA/G and CAA repeats cause predicted differences in the structure of the larger transcript.

### A series of transgenic *Drosophila* strains expressing ataxin-2 bearing different polyQ codon repeat sequences

**Figure 2:**
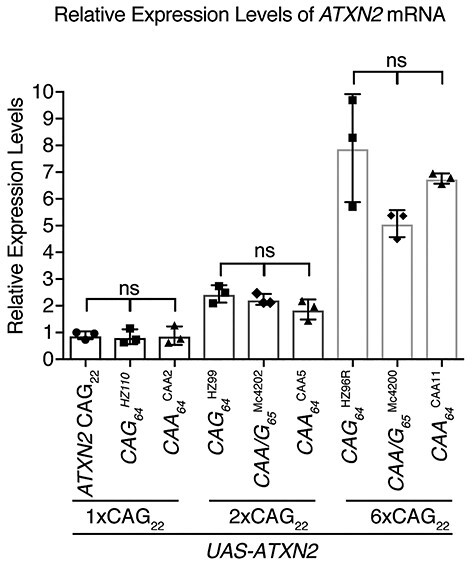
mRNA levels of *UAS-ATXN2* transgenes. *ATXN2* mRNA levels were measured relative to *ATXN2-CAG*_22_ by real-time PCR. The *ATXN2* transgenic lines were expressed by the inducible daughterless gene switch (daGS)*-GAL4* driver. Males of the correct genotype were aged for 48 h on 200 μg of RU486, at 25°C. The abdomens were removed and discarded, and total RNA was isolated from the remaining thorax and head tissue from ~10 males per genotype. Data represent the mean (s.e.m) from three independent cohorts. The transgenic lines are grouped by *ATXN2* mRNA expression levels (1×, 2× and 6×) relative to the *ATXN2-CAG*_22_ mRNA levels. One-way ANOVA with Tukey’s test was performed between repeat length groups. See Supplementary Material, Table S4 for full genotypes.

To measure and compare disease-causing toxicity of human *ATXN2* with an expanded CAG versus CAA/G versus CAA repeat, we generated approximately 10 independent strains of transgenic *Drosophila* (also referred to as the fly) for each repeat type. Each fly line was characterized for repeat length using direct DNA sequencing of the genomic DNA. The *ATXN2* mRNA expression levels were measured by real-time PCR and compared with our previously generated *ATXN2-CAG_22_* fly line (28). This yielded a series of fly lines with specific repeat lengths for each of the distinct repeat sequences and with expression that was equal to twice as high or up to six times greater than *ATXN2*-*CAG_22_* (Fig. 2, Supplementary Material, Fig. S1, Supplementary Material, Table S1). The pure CAG-repeat lines and pure-CAA lines had lengths of 64 units and will be referred to as CAG_64_ and CAA_64_, respectively, whereas the CAA/G repeat lines had a unit length of 65 and is referred to as CAA/G_65_. We selected transgenic *ATXN2* fly lines of the different repeat structures and grouped them into those with expression levels comparable to *ATXN2-CAG_22_* (1×) or levels that were either 2-fold (2×) or 6-fold (6×) greater than *ATXN2-CAG_22_* (Fig. 2, Supplementary Material, Table S1).

**Figure 3:**
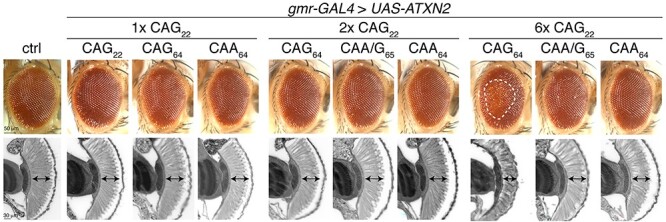
The effects in the eye of ataxin-2 bearing different polyQ repeat sequences. The *ATXN2* transgenes were expressed selectively in the fly eye with the *gmr-GAL4* driver and crosses were raised at 24°C. The external eye (upper panel) and internal retina (lower panel) are presented. Under these conditions, only expression of 6× *ATXN2-CAG_64_* caused visible degeneration of the eye. The white hatched line (upper panel) encompasses the external degeneration and the black double headed arrows (lower panel) indicate retinal width. Control (ctrl) is *w*; *UAS-mCD8-GFP/+*; *gmr-GAL4/+*. See Supplementary Material, Table S4 for full genotypes.

Our selected *ATXN2* transgenes (1×, 2× and 6×) were expressed in the fly eye with *gmr-GAL4* and the resulting effect on the eye was assessed both externally and internally. Our analysis showed that *ATXN2* expressed at lower levels (1× and 2×) did not confer a visible effect on the external eye surface or internal retina (Fig. 3). However, *ATXN2-CAG_64_* (line HZ96R) with expression 6× that of *ATXN2-CAG_22_* (referred to as 6× *ATXN2-CAG_64_*) caused external eye disruption with mild loss of pigmentation and internal thinning of the retina (Fig. 3). Intriguingly, expression of either *ATXN2-CAA/G_65_* or *ATXN2-CAA_64_* with expression 6× that of *ATXN2-CAG_22_* (referred to as 6× *ATXN2-CAA/G_65_* and 6× *ATXN2-CAA_64_*) failed to confer a visible effect on the eye (Fig. 3). Thus, we selected the 6× *ATXN2* fly lines to investigate potential differences between a CAG, CAA/G and CAA encoded repeat expansion.

### A pure CAG-repeat is required for toxicity of ataxin-2 in the fly eye

**Figure 4:**
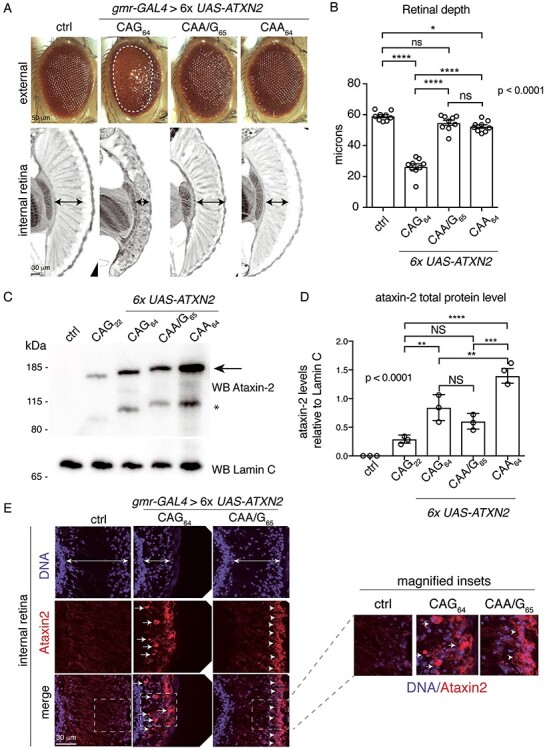
Protein expression of the *ATXN2* transgenes shows the CAA/G_65_ and CAA_64_ express robust protein yet are not toxic. (**A**) Expression of 6× *ATXN2-CAG_64_* at 25°C disrupted the external eye (upper panel) and internal retina (lower panel), while 6× *ATXN2-CAA/G_65_* or 6× *ATXN2-CAA_64_* did not cause any visible degeneration. The white hatched line encompasses external degeneration, and the black double headed arrows indicate retinal width. Expression of 6× *ATXN2-CAG_64_* at 25°C is more toxic than at 24°C (Fig. 3). Control (Ctrl) is *w*; *UAS-mCD8-GFP/+*; *gmr-GAL4/+*. (**B**) Expression of 6× *ATXN2-CAG_64_* in the eye significantly reduced retinal width compared with the control. Mean (s.e.m.) is presented. Each data point represents one head where three independent sections were measured and averaged; this was performed on three animals from three independent biological repeats. The averaged data from each animal examined are presented and were analyzed by one-way ANOVA and Tukey’s test. NS: not significant. Asterisks, significant. ^*^P < 0.05 and ^****^P < 0.0001. Control (Ctrl) is *w*; *UAS-mCD8-GFP/+*; *gmr-GAL4/+*. (**C**) *ATXN2-CAG_22_*, 6× *ATXN2-CAG_64_*, 6× *ATXN2-CAA/G_65_* and 6× *ATXN2-CAA_64_* were expressed by the inducible daGS*-GAL4* driver. Males of the correct genotype were aged for 48 h on 2 μg of RU486, at 25°C. Protein was isolated from head tissue from 10 males per genotype. Upper panel is immunoblotted for ataxin-2, arrow indicates the full-length protein, * indicates the previously reported cleavage product (22). Lower panel is the same immunoblot probed for Lamin C. Control (Ctrl) is *w^1118^*; *+/+*; *+/+ (BL5905).* (**D**) Ataxin-2 protein levels were quantified relative to Lamin C. 6× *ATXN2-CAA_64_* total protein levels were significantly higher than the protein produced by 6× *ATXN2-CAG_64_* and 6× *ATXN2-CAA/G_65_*. Mean (s.e.m.), *n* = 3, one-way ANOVA and Tukey’s test is presented. NS: not significant. Asterisks, significant. ^*^P < 0.05, ^**^P < 0.01 and ^***^P < 0.001. Control (Ctrl) is *w^1118^*; *+/+*; *+/+ (BL5905)*. (**E**) Expression of 6× *ATXN2-CAA/G_65_* in the eye with the *gmr-GAL4* driver leads to diffuse protein localization in the retina (arrowheads). In contrast, 6× *ATXN2-CAG_64_* when expressed in the eye accumulates as aggregated inclusions (arrows). Control (Ctrl) is *w*; *UAS-mCD8-GFP/+*; *gmr-GAL4/+.* See Supplementary Material, Table S4 for full genotypes.

Our initial analysis indicated that only the 6× *ATXN2-CAG_64_* line conferred retinal degeneration. We examined this in greater detail by quantifying the eye degeneration caused by expression of the 6× *ATXN2-CAG_64_* transgene compared with the 6× *ATXN2-CAA/G_65_* and 6× *ATXN2-CAA_64_* transgenes. Our analysis showed that expression of the 6× *ATXN2-CAG_64_* transgene resulted in a significant decrease (*P* < 0.0001) in retinal width, indicating degeneration (Fig. 4A and B). In contrast, expression of the 6× *ATXN2-CAA/G_65_* and 6× *ATXN2-CAA_64_* transgenes caused little to no retinal degeneration (Fig. 4A and B). To confirm that expression of *ATXN2-CAG_64_* at a level of 6× the *ATXN2-CAG_22_* line conferred toxicity, we expressed a combination of multiple independent *ATXN2-CAG_64_* transgenic lines in the eye, which combined would be 6× *ATXN2-CAG_22_*, and confirmed both external and internal degeneration of the fly eye (Supplementary Material, Fig. S2). These data indicate that the degeneration of the fly eye is due to expression of *ATXN2-CAG_64_* at a level that is 6× *ATXN2-CAG*_22_.

We next considered that the different *ATXN2* transgenic lines, despite similar mRNA expression levels (see Fig. 2), may be translated to different extents, and perhaps, the 6× *ATXN2-CAG_64_* fly line had higher levels of the ataxin-2 protein compared with the 6× *ATXN2-CAA/G_65_* and 6× *ATXN2-CAA_64_* fly lines. To examine this possibility, we assessed the levels of the ataxin-2 protein in fly heads by western immunoblot. These data showed that the level of ataxin-2 protein expressed from the 6× *ATXN2-CAG_64_* and 6× *ATXN2-CAA/G_65_* transgene did not significantly differ and, consistent with our mRNA analysis in Figure 2, both were higher than *ATXN2-CAG_22_* (4 ± 2 fold and 3 ± 2 fold (SD), respectively) (Fig. 4C and D). However, strikingly, despite conferring no toxicity when expressed in the fly eye (Fig. 4A and B), the level of ataxin-2 protein expressed by the 6× *ATXN2-CAA_64_* line was significantly higher than the ataxin-2 protein expressed by *ATXN2-CAG_22_*, 6× *ATXN2-CAG_64_* and 6× *ATXN2-CAA/G_64_* (Fig. 4C and D). Thus, our data suggest that the toxicity associated with ataxin-2 is not simply due to expression levels of an ataxin-2 protein with a long polyQ Q64/Q65 domain but is also due to the presence of a pure and expanded CAG repeat sequence in the *ATXN2* mRNA.

To further explore the differences in the ataxin-2 protein produced by the different *ATXN2* transgenes, we examined ataxin-2 protein localization in fly retinal tissue. This revealed that ataxin-2 when expressed by the 6× *ATXN2-CAG_64_* transgene accumulated into punctate aggregates (Fig. 4E). In contrast, ataxin-2 expressed from the 6× *ATXN2-CAA/G_65_* (Fig. 4E), or *ATXN2-CAA_64_* transgene (Supplementary Material, Fig. S3) showed a diffuse expression pattern. Collectively, our data indicate that a CAG-repeat expansion in *ATXN2* is more toxic than the interrupted CAA/G-repeat and CAA-repeat and that the *ATXN2-CAG_64_* mRNA promotes the aggregation of the ataxin-2 protein. Combined, our data suggest that at the levels and duration of ataxin-2 expression used in our studies, RNA toxicity is a component of the SCA2 mutation in ataxin-2.

### Differential toxicity in the nervous system of CAG versus CAA/G versus CAA *ATXN2* transgenes

**Figure 5:**
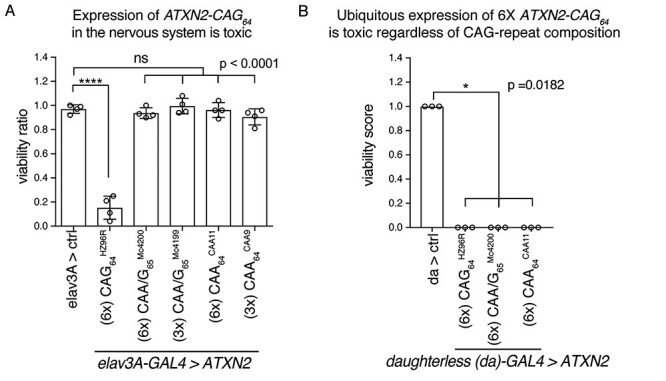
Differential tissue toxicity of *ATXN2* transgenes. (**A**) The *ATXN2* transgenes were expressed selectively in the nervous system with the *elav3A-GAL4* driver. The resulting progeny were scored for genotype and a ratio was calculated based on the expected Mendelian frequencies. Only *ATXN2-CAG_64_* was toxic in the nervous system. Mean (s.e.m.), *n* = 4 experiments, one-way ANOVA and Tukey’s test is presented. NS: not significant. Asterisks, significant. ^***^P < 0.001. Control (Ctrl) is *w*; *elav3A-GAL4/UAS-mCD8-GFP*; *+/+*. (**B**) Ubiquitous expression of the *ATXN2* transgenes with *daughterless-GAL4* (*da-GAL4)* is toxic regardless of the sequence composition of the polyQ repeat. Flies were scored as viable (1) or completely inviable (0), each datapoint represents a biological repeat (*n* = 3 experiments). Data were analysed using a Kruskal–Wallis test and a Dunn’s multiple comparison test. Control (Ctrl) is *w*; *UAS-mCD8-GFP/+*; *da-GAL4/+*. See Supplementary Material, Table S4 for full genotypes.

Our analyses in the *Drosophila* eye indicated that only the 6× *ATXN2-CAG_64_* transgene conferred toxicity when expressed in the eye with the *gmr-GAL4* driver (see Fig. 4). We next set out to determine if the differential toxicity of a pure CAG repeat versus a CAA/G-repeat or a CAA repeat extended to other tissue types. To do this, we expressed the *ATXN2* transgenes in a range of different fly tissues. To test the effect of the repeat in the context of the nervous system, we expressed the *ATXN2* transgenes with the *elav3A-GAL4* driver, which expresses in all neurons of the brain from early developmental stages through to adulthood. Consistent with expression of the 6× *ATXN2-CAG_64_* transgene conferring toxicity to the eye, expression of the 6× *ATXN2-CAG_64_* transgene selectively in neurons was highly toxic and caused a developmental lethality that resulted in very few progeny surviving to adulthood (Fig. 5A). The 6× *ATXN2-CAA/G_65_* and 6× *ATXN2-CAA_64_*, as well as 3× *ATXN2-CAA/G_65_* and 3× *ATXN2-CAA_64_* (Supplementary Material, Fig. S3), conferred little or no toxicity when expressed in the nervous system (Fig. 5A). Thus, the selective toxicity of the 6× *ATXN2-CAG_64_* transgene to the retina was a shared property with expression in the entire nervous system. Next, we directed the expression of the *ATXN2* transgenes ubiquitously in the animal from early development using the *daughterless (da)-GAL4* driver. Intriguingly, in this case, expression of all three 6× *ATXN2* transgenes (CAG_64_, CAA/G_65_ and CAA_64_) was toxic and led to developmental lethality that resulted in no adult survivors (Fig. 5B). Furthermore, ubiquitous expression of *ATXN2-CAG_22_*, *ATXN2-CAG_32_* and the 1× expanded repeat proteins (*ATXN2-CAG_64_*, *ATXN2-CAA/G_64_* and *ATXN2-CAA_64_*) also resulted in no adult survivors (Supplementary Material, Table S2). These findings suggest that ataxin-2 toxicity when expressed ubiquitously may be associated with the ataxin-2 protein versus the specific mRNA sequence of the polyQ domain.

### The enhancement of TDP-43 toxicity by ataxin-2 is mediated by the protein and not the mRNA

**Figure 6:**
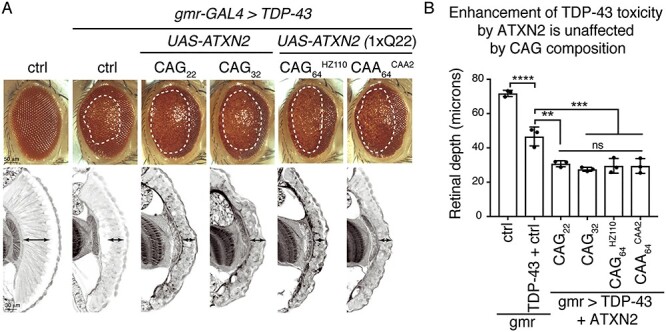
The ataxin-2 protein with a CAG or CAA encoded polyQ enhances TDP-43 toxicity. (**A**) The toxicity of TDP-43 on the external (upper panel) and internal (lower panel) eye is enhanced by the co-expression of *ATXN2-CAG_22_* and *ATXN2-CAG_32_* seen by the degenerate eye and collapsed retina. TDP-43 toxicity in the external and internal eye was equally enhanced by the co-expression of *ATXN2-CAG_64_* or *ATXN-CAA_64_*. White hatched lines (upper panel) indicate external degeneration and double-headed arrows (lower panel) indicate retinal width. Control is *w*; *mCD8-GFP/+*; *gmr-GAL4/+.* (**B**) Quantitation of the retinal depth confirms that all *ATXN2* transgenes, regardless of repeat sequence, show similar impact on retinal depth in this assay. See Supplementary Material, Table S4 for full genotypes.

ALS patients with an intermediate CAA/G expansion in *ATXN2* present with the pathological hallmark of cytoplasmic TDP-43 aggregates in affected neurons (40,44), indicating that mutation in *ATXN2* impacts ALS disease features. Furthermore, we previously demonstrated that upregulation of *ATXN2-CAG_22_* and *ATXN2-CAG_32_* in the fly enhances the toxicity of the wild-type form of TDP-43 (28). We thus determined whether the *ATXN2* transgenes with a longer CAG repeat length enhanced the toxicity of the wild-type TDP-43 and whether the composition of the repeat (CAG or CAA) altered the enhancement. We selected *ATXN2-CAG_64_* and *ATXN2-CAA_64_* transgenes that expressed *ATXN2* mRNA at levels that did not significantly differ from *ATXN2-CAG_22_* and were classified as 1× (see Fig. 2, Supplementary Material, Table S1). Co-expression of the *ATXN2* transgenes with TDP-43 in the fly eye showed that the 1× *ATXN2-CAG_64_* and 1× *ATXN2-CAA_64_* transgenes enhanced TDP-43 degeneration of the external eye and internal retina in a manner similar to that of *ATXN2-CAG22* (Fig. 6A and B and Supplementary Material, Fig. S4). These data are consistent with our previous findings that the interaction between TDP-43 and ataxin-2 with an intermediate CAG repeat is at the protein level (26,28), indicating that the RNA sequence of the ataxin-2 repeat appears not to influence the interaction with TDP-43. These data suggest that RNA toxicity arising from the pure CAG-repeat in *ATXN2* is a feature of SCA2, but not ALS.

### Regulation of *ATXN2*-*CAG*_*64*_ toxicity by proteins involved in transcription and translation

Mounting evidence indicates that transcription of long-repeat sequences, such as CAG and GGGGCC, in the context of short fragments of coding sequence is dependent on specific transcription factors (45–48). Furthermore, the transcribed RNA-repeat sequence can initiate RAN translation to generate peptides that are toxic (10–12,14,15,17,49). These findings suggest that targeting pathways that can selectively inhibit the transcription and translation of long repeat sequences may have potential as a therapeutic approach. Given that a pure repeat of CAG_64_ in the context of the full-length coding sequence for *ATXN2* (6× *ATXN2-CAG_64_*) conferred strong toxicity to the fly eye, we considered that transcriptional and RAN translational mechanisms linked to the CAG repeat may be involved. Previously, we defined a number of gene modifiers important for transcription of an expanded GGGGCC-repeat sequence that is found in the *C9ORF72* gene in ALS and frontotemporal degeneration (FTD) (46,47). Those studies indicated that the DRB Sensitivity Inducing Factor (DSIF) and polymerase-associated factor 1 (PAF1) complex are important for transcription through the highly structured GGGGCC-repeat region. The DSIF complex has also been shown to be important for transcription of CAG repeats in the context of the Huntington’s disease fragment proteins in yeast and mice (45,50).

**Figure 7:**
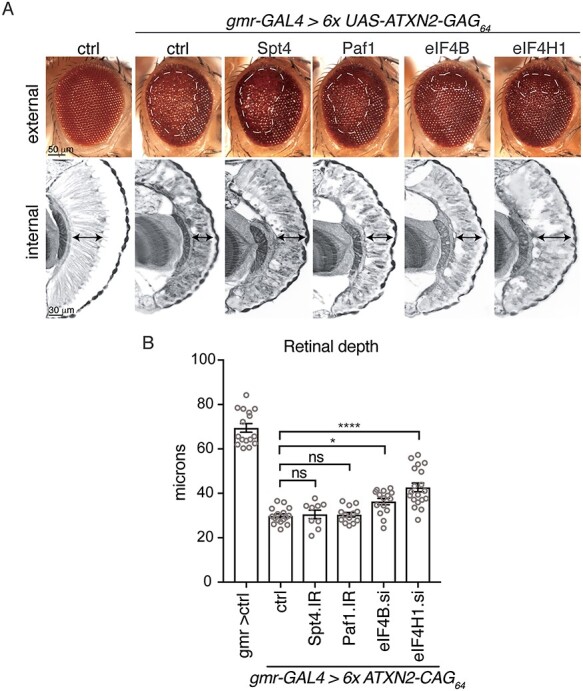
Interactions between *ATXN2-CAG_64_* and translation factors that modulate GGGGCC repeat toxicity. (**A**) *ATXN2-CAG_64_* was co-expressed in the eye with either inverted repeats or siRNAs (si) directed to a control (ctrl), the Paf1 complex (Spt4 or Paf1) or to potential RAN translation factors (eIF4B or eIF4H1). The effect of the PAF1 complex or potential RAN translation factors on *ATXN2-CAG_64_* toxicity and the resulting effect on toxicity was assessed in the external eye (white hatched line, upper panel) and internal retina (double-headed arrow, lower panel). Control (ctrl) is *w*; *gmr-GAL4/+.* (**B**) Quantification of internal retinal width showed that only downregulation of eIF4H1 and eIF4E3 significantly improvement retinal width compared with the control (ctrl). Mean (s.e.m.), one-way Anova and a Dunnett’s test. ^***^P < 0.0001, ^*^P < 0.05, ns not significant. Control (ctrl) is *w*; *gmr-GAL4/+.* See Supplementary Material, Table S4 for full genotypes.

To determine if the DSIF complex or the PAF1 complex regulated the toxicity of *ATXN2-CAG_64_*, we determined whether the mis-regulation of two crucial components of each complex (Spt4 and Paf1, respectively) altered the eye degeneration caused by *ATXN2-CAG_64_*. These studies showed that downregulation of either Spt4 or Paf1 had little to no effect on *ATXN2-CAG_64_* associated eye toxicity (Fig. 7), despite their robust effects on the GGGGCC repeat in the fly (46,47). These data indicate that the DSIF and PAF1 transcriptional protein complexes do not impact the toxicity of the expanded CAG repeat in the context of the ataxin-2 mRNA.

We next addressed the involvement of RAN translation in the observed toxicity arising from the 6× *ATXN2-CAG_64_* transgene. Previously, we discovered in the context of the GGGGCC-expanded repeat, elongation factors eIF4B and eIF4H function critically *in vivo* for production of GR peptides from the GGGGCC repeat (49). We therefore determined whether either eIF4B or eIF4H were critical for the eye toxicity generated by the CAG_64_ repeat in *ATXN2*. Our data indicated that reduction of eIF4B or eIF4H had some effect to mitigate the toxicity of the 6× *ATXN2-CAG_64_* transgene (Fig. 7). These data raise the possibility that disease-associated toxicity of the expanded CAG repeat in the *ATXN2* mRNA may involve translational mechanisms and share some mechanistic overlap with RAN translation of the non-coding GGGGCC repeat.

## Discussion

Here, we have generated and characterized a range of novel fly models of ataxin-2-associated neurodegenerative disease. Our ataxin-2 transgenic fly models express a pathogenic polyQ ataxin-2 protein encoded by different glutamine-coding codons: the SCA2-associated CAG repeat, interrupted CAA/G repeat associated with parkinsonism and ALS, or an experimentally produced pure CAA repeat. Altering the composition of the CAG repeat is predicted to change the RNA structure, with only the CAG repeat forming a long single hairpin (see Fig. 1). Strikingly, only the transgene with a polyQ domain encoded by the pure CAG-repeat showed a degenerative effect when expressed in the eye or nervous system (see Figs 3–5). The difference was not due to differing levels of the ataxin-2 protein since the transgene with a CAA (*ATXN2-CAA_64_*) sequence showed significantly higher levels of the protein than the *ATXN2-CAG_64_* transgene. Furthermore, the interrupted *ATXN2-CAA/G_64_* repeat transgene, which is predicted to fold into multiple shorter hairpins, produced protein at levels no different to the *ATXN2-CAG_64_* transgene yet still conferred no toxicity in the eye or nervous system in our study (see Figs 3–5). Intriguingly, the ataxin-2 protein, despite being expressed at similar levels with the different repeat sequences, showed an aggregated pattern of expression from the CAG-encoded transgene, compared with the CAA/G or CAA transgenes (see Fig. 4). These findings indicate that abolishing the long CAG hairpin (CAA repeat) or replacement of a long hairpin with several shorter hairpins (CAA/G) is sufficient to prevent disease-associated toxicity to the eye and nervous system of the fly. All repeat variants (CAG, CAA/G and CAA) of *ATXN2* were toxic when expressed in the entire animal (see Fig. 5) indicating that the nervous system appears more sensitive to the expanded CAG repeat bearing *ATXN2* transgene. One potential mechanism underling the CAG-induced toxicity may be that a pure CAG could encode toxic proteins in alternative reading frames through RAN translation or through frameshifting. Such proteins may be more aggregation prone than ataxin-2, thus leading to the protein aggregation that we observe with the CAG-repeat encoded ataxin-2 protein. Our data indicate that the toxicity of the 6× *ATXN2-CAG_64_* transgene shows sensitivity to the levels of a translation factor (eIF4H; see Fig. 7) that also modulates the toxicity of a GR peptide from the GGGGCC repeat (49). The RNA hairpin formed by a pure CAG repeat could also convey toxic features, such as activating the dsRNA pathway, among others (51–53). Collectively, these studies provide a novel fly model for dissecting different pathogenic mechanisms of ataxin-2-associated neurodegenerative disease.

### The human *ATXN2* gene has different nucleic acid sequences of the polyQ domain and different clinical manifestations

PolyQ repeat expansions in ataxin-2 are interesting in that they are a risk for a number of different clinical presentations. Uninterrupted CAG repeat expansions in *ATXN2* (34 and greater) present with SCA2, which is characterized by cerebellar dysfunction and ataxia (2,21,54,55). In contrast, expansions greater than the normal 22/23, but typically below the threshold for SCA2 (>33), can present with the motor neuron disease ALS (26,38). CAA interrupted repeat expansions of SCA2 length can present with parkinsonism (36,56), which is a movement disorder characterized by tremors and stiffness. Interrupted expansions have also been associated with FTD (35). These different disease presentations reflect the varying extent to which different brain regions are affected, with differing penetrance of functional loss in different brain regions presumably underlying symptomatic presentation of ataxia versus motor neuron degeneration versus parkinsonism versus dementia as the dominant feature. Thus, a fascinating aspect of these different ataxin-2-associated disease presentations is that the domain encoding the polyQ is an uninterrupted CAG repeat for SCA2, whereas ALS, parkinsonism and FTD present with CAA interrupted CAG repeats (37,40,56). For the polyQ diseases, the purity of the repeat influences its tendency to expand both intergenerationally and somatically (57–60). As a pure uninterrupted CAG repeat disease, SCA2 is predicted to be associated with greater somatic expansions, and thus a more toxic protein in tissues that bear the expansions. In the fly, pure CAG repeats can expand intergenerationally, although at a much lower frequency than in humans and somatic expansion is extremely rare (61). Thus, the differing toxicity of the 6× *ATXN2-CAG_64_* in the fly is not likely due to changes in the length of the repeat; rather, other biological features associated with pure versus interrupted CAG repeats likely underlie the differential toxicity seen in the fly.

### Biological processes associated with pure versus interrupted repeats

The structure of an RNA comprised of pure CAG repeats is a hairpin (42,62). Such hairpins may sequester RNA-binding proteins, leading to the loss of function of the sequestered protein from other activities in the cell (5,63). In contrast, an RNA that is CAA interrupted is predicted to undertake a branched structure (see Fig. 1) and thus may not sequester proteins or not sequester them to the same extent. The CUG-repeat expanded hairpin that is associated with the myotonic dystrophy protein kinase has been shown to activate signaling pathways (51,64), highlighting another feature that may be associated with pure hairpin repeats. Thus, by sequestering RNA-binding proteins and/or by activating select pathways, the 6× *ATXN2-CAG_64_* transgene may be more toxic than an RNA expressed from a transgene bearing an interrupted CAA/G-repeat RNA or the pure CAA-repeat RNA.

Pure CAG-repeat sequences, if sufficiently long, have the capacity to frameshift (65,66) or to undergo RAN translation (12,13,67). Thus, a long CAG repeat could frameshift or encode poly-alanine (A) and poly-serine (S) protein. Both polyA and polyS have been shown to be toxic to neurons in culture, and potentially more toxic than polyQ (65,66). Previous studies with an *in vivo* fly model for the intronic repeat expansion associated with ALS/FTD of GGGGCC highlighted specific translation factors that are important for expression of a poly-GR peptide (48,49). Among these, eIF4B and eIF4H were key to GGGGCC toxicity and importantly reducing their function on their own has little effect on the animal. Here, we found that reduced expression of eIF4B and eIF4H also mitigates toxicity of the 6× *ATXN2-CAG_64_* transgene. These factors can stimulate the helicase activity of eIF4A for translation of structured RNAs, indicative of a role in RAN translation. Our data are consistent with a previous study in mammalian cells that showed that RAN translation can occur from an expanded CAG repeat that contained a short stretch of the downstream *ATXN2* sequence (17). Interestingly, here, we found that the transcriptional regulator Spt4 appears not to impact the toxicity of the expanded CAG repeat in the *ATXN2* transgene, although it has been shown to be important for transcription of pathogenic CAG-repeats in Huntington’s disease transgenes and the GGGGCC repeat of ALS/FTD (45,46,50). We also did not observe *ATXN2-CAG_64_* to be sensitive to PAF1, a transcription factor that impacts toxicity of the GGGGCC repeat (47). These findings may indicate that in our *ATXN2-CAG_64_* fly model, RAN translation rather than repeat-associated transcription, has a bigger influence on toxicity. Emerging data, including this study, indicate some RAN translation factors are important for more than one type of nucleotide repeat expansion, such as GGGGCC, CGG and CAG-repeat expansions (14,15,49,68–71). A fuller understanding of RAN translation is required to appreciate the biological overlap between different repeat expansion sequences.

Intriguingly, the 6× *ATXN2-CAG_64_* was highly toxic in the retina and nervous system compared with the interrupted CAA/G repeat or CAA repeat; however, all transgenes were equally toxic when expressed ubiquitously (see Figs 4 and 5). These findings suggest that the brain and nervous system have selective processes important for toxicity of a pure CAG repeat. The similar toxicity observed for the three repeat types when broadly expressed may be due to expression of ataxin-2 and not processes associated with a pure CAG-repeat RNA encoding the polyQ domain. Notably, the CAA- and CAG-repeat encoded *ATXN2* similarly enhanced TDP-43 (see Fig. 6), underscoring that the interaction between TDP-43 and ataxin-2 is at the protein level, and the CAG purity of the Ataxin-2 polyQ repeat has minimal impact on this interaction.

### Concluding remarks

We have uncovered a differential effect of a CAG-repeat encoded ataxin-2 protein versus the same protein encoded by an interrupted CAA/G repeat. Additional study of this system, including for gene interactions and directed analysis to test mechanisms (for example, mechanisms of RAN translation), may help to uncover specific pathways and gene players that contribute to the different clinical manifestations associated with the CAG repeat composition in *ATXN2* in human disease. These players may also help reveal additional mechanisms associated with the broader repeat expansion diseases.

## Materials and Methods

Key reagents and sources are listed in Supplementary Material, Table S4.

### *Drosophila* culture and lines

Fly stocks were maintained on standard cornmeal molasses agar. Progeny from fly crosses were raised at the indicated temperatures. *UAS-ATXN2(CAG)_64_*, *UAS-ATXN2 (CAA/G)_65_* and *UAS-ATXN2 (CAA/G)_64_* transgenic lines were generated by The BestGene, Inc (Chino Hills, CA). The control transgene was y^1^ w*; UAS-mCD8-GFP, which was obtained from the Bloomington *Drosophila* Stock Center and backcrossed into *w^1118^* (stock BL5905) with the *y^1^* removed from the genotype. The *daughterless(da)-GAL4* was obtained from Bloomington *Drosophila* Stock Center. *Elav3A-Gal4* was a gift of M. Tanouye (72). *Glass multimer reporter gmr-GAL4(III)* was a gift from Y. Hiromi*.* The *UAS-TDP-43, UAS-ATXN2-Q22* and *UAS-ATXN2-Q32* are described (26,28)*.* Sources and genotypes of fly lines are given in Supplementary Material, Table S4. Experimental crosses were carried out at 25°C unless stated otherwise. For all experimental crosses, the internal temperature of the incubator was routinely monitored.

### Ataxin-2 transgenes with variable sequence repeats

Transgenes expressing *ATXN2* with a CAA/G interrupted repeat were generated as follows. Two oligos were synthesized, 65caag-1 and 65caag-2 (Supplementary Material, Table S3). Two PCR reactions were set up using Phusion™ DNA polymerase (ThermoFisher Scientific, Waltham, MA) with *pUAST-ATXN2-(CAG)22* as template, one with primer set NB1781 and 65caag-1 and the second with primer set 65caag-2 and NB1792 (Supplementary Material, Table S3). The PCR products were gel purified, phosphorylated with T4 polynucleotide Kinase (NEB M0201S) and ligated with Quick ligation kit (Roche 11 635 379 001). A PCR reaction was set up using Phusion DNA polymerase with the above ligation reaction as template with primers NB1781 and NB1792. The PCR product was gel purified, subcloned into the pGEMT vector (Promega). The resulting colonies were prepared for sequencing to determine the sequence and repeat length of the various colonies. The clone with the desired repeat length was digested with *Acs*I and *Xho*I, gel purified, ligated with pUAST-ATXN2 digested with *Acs*I/*Xho*I. The colonies were sequenced to confirm the final construct pUAST-ATXN2-CAA/G_65_. The same strategy was used to generate pUAST-ATXN2-CAA_64_, except using primers CAA64-1A and CAA64-2S (Supplementary Material, Table S3). pUAST-ATXN2-CAG_64_ made from a human *ATXN2* clone with a long polyQ repeat [generously shared by S. Pulst (University of Utah, Salt Lake City, UT)], the final insert was sequence verified. The constructs were maxi-prepped and transformed into *Drosophila* (The Best Gene, Inc., Chino Hills, CA). Independent transgenic insertions were mapped and balanced to the chromosomes. To determine the repeat lengths in individual transgenic fly lines, genomic DNA was isolated from single animals. PCR was performed using primers Sca2-S2 and Sca2-B (Supplementary Material, Table S3) with Takara LA taq polymerase (Takara RR02AG). The PCR products were run on a bioanalyzer to size the repeat.

### Realtime PCR

Approximately 10–20 males per genotype were aged on fly food containing 200 μg of RU486 (Sigma-Aldrich, M8046) for 48 h. Biological triplicates were collected for each genotype. RNA isolation and real-time PCR were performed as previously described (73), with minor alterations. The abdomen from the RU486-treated males was removed and discarded, and the remaining tissue from 10 males per genotype was homogenized by hand in 250 μl of Trizol (ThermoFisher Scientific, 15 596 026). After adding an additional 250 μl Trizol, the RNA was extracted with chloroform and precipitated with ethanol and sodium acetate, pH 5.2. RNA was re-suspended in RNase-free water. RNA quality was assessed by Bioanalyzer. Genomic DNA was removed from the total RNA using Turbo DNA-free (Amersham, AM1907). Random primed cDNA was made from 250 μg of RNA using Superscript III (ThermoFisher Scientific). Real-time PCR was performed using SYBR FAST (Amersham 4 385 610), with all samples and replicates run on the same 384-well plate. The ΔΔCt method was used to determine mean fold change. Primers used were SV40 FP2, SV40 RP2, β-Tubulin FP and β-tubulin RP (Supplementary Material, Table S3).

### External eye microscopy, paraffin sectioning and quantification

For external eye microscopy, three female flies were imaged with a Leica Z16 Apo A microscope, DFC420 camera and 1.0× planapochromatic objective (115× magnification and 0.117 numerical aperture) along with Leica Application Suite Montage module software. For paraffin sections, fly heads were fixed in Bouin’s solution (Sigma-Aldrich, HT10132) for 6d at RT and then leached in 1 M Tris–HCl, pH 8.0 and 8.7% NaCl (Thermo Fisher, 15 568–025) overnight. The tissue was incubated in a series of ethanol washes each for 30 min with agitation (70% EtOH, 90% EtOH, 95% EtOH, 95% EtOH, 100% EtOH, 100% EtOH), incubated twice in xylene for 1 h each and finally incubated twice in paraffin (Leica Biosystems) at 60°C for 1 h. Heads were mounted into wax molds and 8 μm paraffin sections were cut in the horizontal plane and mounted onto glass slides. Three heads of the same genotype were collected on each slide. Tissue was visualized using the autofluorescent property of the fly brain with a Leica DMRA2 microscope, DC500 camera, HC PLAN APO objective lens (20× magnification and 0.70 numerical aperture) and 1.6× tube lens, along with Leica FireCam 1.2.0 software. Sections for quantification were imaged at the same anatomical level of the brain, which was where the antennal nerve connects to the antennal lobe; three adjacent sections at this anatomical level were captured for each head. Retinal depth was measured by drawing a line from lower edge of the retina (proximal to the optic lamina) out toward the lens. The line drawn and measured was always in line with the crossover point of the optic chiasm that was visible in the optic lobe. For quantification, three females were imaged per genotype. The experiments were repeated three times independently. Images are presented in reverse black and white. Retinal depth in internal eye images was quantified using ImageJ (https://imagej.nih.gov/ij/). One-way ANOVA and Tukey’s multiple comparisons were performed with a significance threshold of *P* < 0.05.

### Western immunoblots

Immunoblots on fly head tissue were performed as previously described with minor alterations (74). Approximately 20 females per genotype per repeat were aged on fly food for 48 h containing 200 μg RU486 (Sigma-Aldrich, M8046) (100 μl of 2 mg/ml RU486 in 200-proof ethanol). The heads from 10 female flies per genotype were collected and homogenized in 100 μl of protein buffer containing 100 mM Tris–HCl, pH 7.4, 1 mM EGTA, pH 8, 1× Halt protease inhibitor cocktail (Thermo Fisher, 78 430), 0.5% sodium deoxycholate, 0.5% SDS and 1% NP-40. To each tube, 100 μl of 4× LDS sample buffer (Thermo Fisher, NP0007) with 5% beta-mercaptoethanol. Samples were heat denatured at 95°C for 5 min, chilled on ice for 5 min and centrifuged at 4500 × g for 5 min at 4°C. Care was taken to pipette from the surface of the centrifuged liquid a volume equivalent to 0.25 heads and loaded into each well of a 4–12% Bis-Tris Protein Gel, 1.5 mm along with 10 μl Hi-Mark Unstained Protein Standard (Thermo Fisher, LC5688). Samples were electrophoresed for 2 h 15 min at 115 V in NuPAGE SDS MOPS running buffer (Thermo Fisher, NP0001) and transferred onto a 0.45 μm nitrocellulose membrane by wet transfer in NuPAGE Transfer Buffer (Thermo Fisher, NP0006) with 10% methanol for 75 min at 30 V. The membrane was blocked in 5% non-fat milk (LabScientific, M0841) for 1 h at room temperature. Membranes were incubated in mouse Ataxin-2 primary antibody (1:600 in TBS with 0.05% Tween 20; BD Biosciences, 6 113 378) or in mouse Lamin C primary antibody (1:1000 in TBS with 0.05% Tween 20; DSHB, LC28.26-s) at 4°C overnight with rocking. Membranes were washed in TBS with 0.05% Tween 20 for 5 min, 5 times, at RT with agitation and incubated with goat anti-mouse HRP secondary antibody (1:5000 in TBS with 0.05% Tween 20, Abcam, ab6789) for 1 h at RT, with rocking. Membranes were washed in TBS with 0.05% Tween 20 for 5 min, 5 times, at RT with agitation and incubated in ECL Prime Western Blotting Detection Reagent (Amersham, RPN2232) for 5 min at RT in the dark and detected by chemiluminescence using a GE Healthcare Amersham Imager 600. Ataxin-2 band signal intensity was quantified using ImageJ (https://imagej.nih.gov/ij/) and was normalized to the signal intensity of respective Lamin C loading control bands. One-way ANOVA and Tukey’s multiple comparisons were performed with a significance threshold of *P* < 0.05.

### Cryosections

Cryosections and immunostaining were performed as previously described (75). Adult heads of appropriate genotype were embedded in O.C.T. (Tissue-Tek), 12 μm serial sections were cut and collected on slides. Tissue was fixed in 4% paraformaldehyde in PBS, then stained with appropriate antibodies and Hoechst (0.5 μg/ml for 5 min). Images were scanned, using identical parameters across genotypes, on a Leica confocal microscope. Antibodies used were mouse anti-ATXN2 (1:200; BD611378, BD Biosciences, Billerica, MA), rabbit anti-ATXN2 (1:200; HPA 018295, Sigma-Aldrich, St. Louis, MO), Alexa goat anti-mouse AF568 (1:250; A11036, Invitrogen, Carlsbad, CA) and Alexa goat anti-rabbit AF568 (1:250; A11011, Invitrogen, Carlsbad, CA). Immunostaining with the rabbit antibodies is presented in the figures.

### Viability assays

*UAS-ATXN2-CAG_64_* (6×), *UAS-ATXN2-CAA/G_65_* (6×), *UAS-ATXN2-CAA_64_* (6×) and *UAS-mCD8-GFP* were crossed to either the *da-GAL4* or *elav3A-GAL4* driver lines. All surviving progeny of the expected genotypes were counted every 2 d. The ratio of the actual numbers of the desired genotype divided by the expected numbers of the desired genotype was calculated. The expected numbers were based on the presumption that all genotypes were equally likely to occur. More than 100 animals per genotype were counted for those with regular viability.

## Supplementary Material

McGurk_Rev_Sup_v2_ddab148
