# Supplementary material for: Toxicity of pathogenic ataxin-2 in Drosophila shows dependence on a pure CAG repeat sequence
Source: Hum Mol Genet. 2021 Jun 2;30(19):1797–810. doi: 10.1093/hmg/ddab148 (PMC8444453; doi:10.1093/hmg/ddab148)
Supplement: McGurk_Rev_Sup_v2_ddab148 [file mcgurk_rev_sup_v2_ddab148.pdf]

SUPPLEMENTARY MATERIAL FOR:

Toxicity of pathogenic ataxin-2 in *Drosophila* shows dependence on a pure CAG repeat sequence

Leeanne McGurk[1,2], Olivia M. Rifai[2], Oksana Shcherbakova[2],
Alexandra E. Perlegos[3], China N. Byrns[3,4], Faith R. Carranza[2],
Henry W. Zhou[2], Hyung-Jun Kim[2@], Yongqing Zhu[2] &
Nancy M Bonini [2,3]*

[1] Division of Cell & Developmental Biology, School of Life Sciences,
University of Dundee, Dundee, Scotland, UK
[2] Department of Biology, University of Pennsylvania, Philadelphia, PA USA
[3] Neurosciences Graduate Group and [4]Medical Sciences Training Program,
Perelman School of Medicine, University of Pennsylvania, Philadelphia, PA USA

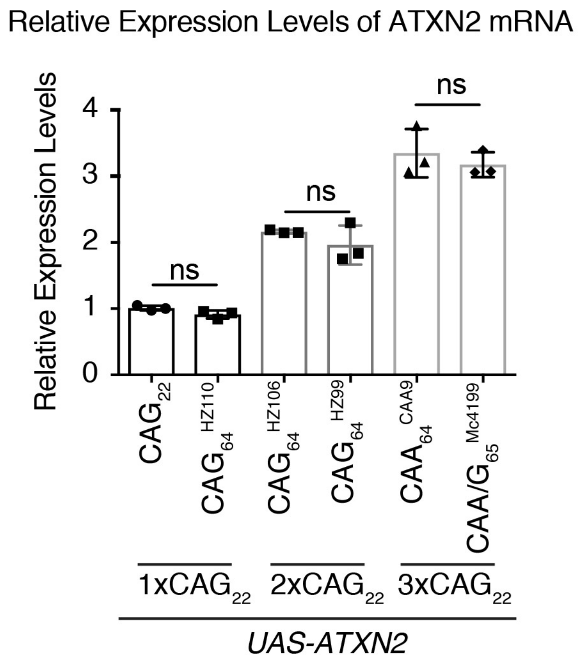

Supplementary Figure S1: mRNA levels of *UAS-ATXN2* transgenes.
Real-time PCR was used to measure *ATXN2* mRNA levels relative to *ATXN2-CAG*$_{22}$. *ATXN2* was expressed by the inducible *daughterless gene switch* (daGS)-*GAL4* driver. Males were aged for 48 hr on 200 µg of RU486, at 25°C, the abdomens were removed and discarded, and total RNA was isolated from the thorax and head tissue from ~ 10 males per genotype. Data represents the mean (s.e.m) from 3 independent cohorts. The transgenic lines are grouped by *ATXN2* mRNA expression levels (1x, 2x, and 3x) relative to the *ATXN2-CAG*$_{22}$ mRNA levels. One-way ANOVA with Tukey's test was performed between repeat length groups.
See Supplementary Table S4 for full genotypes.

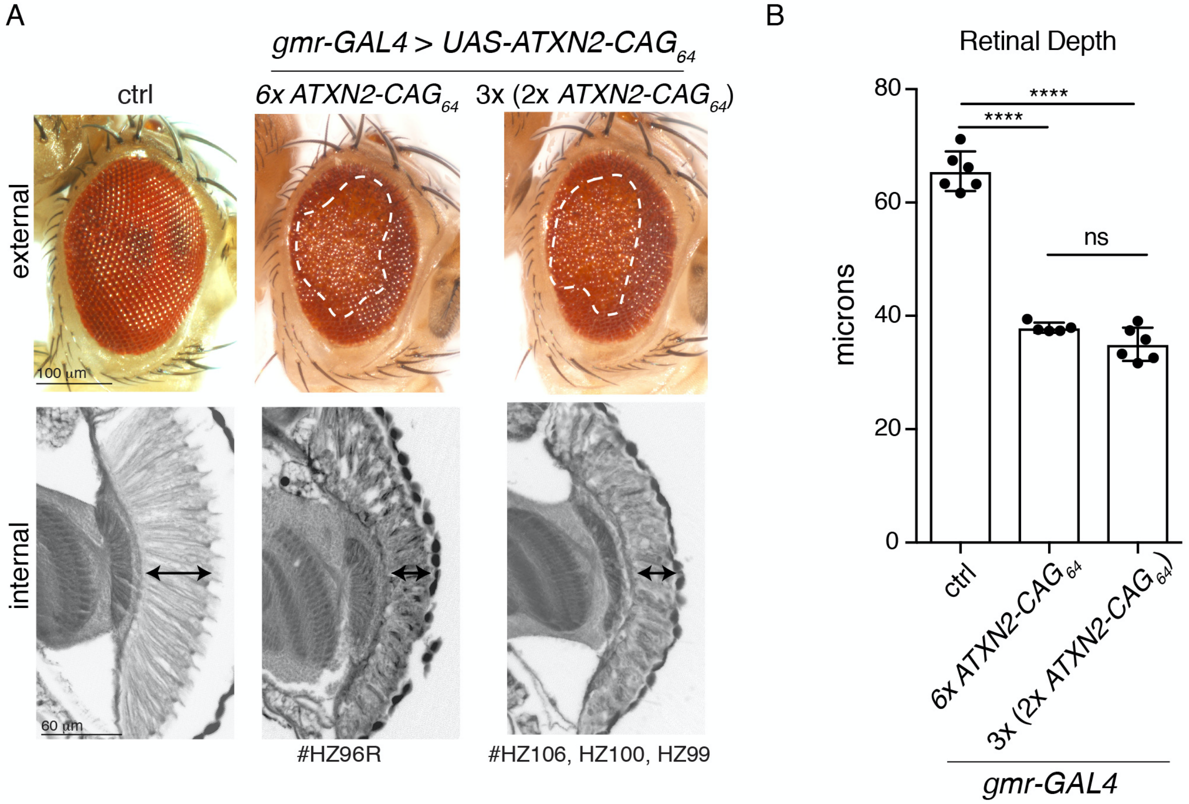

Supplementary Figure S2: Expression of *ATXN2-CAG$_{64}$* is toxic to the eye.

A: Three independent transgenes that express *ATXN2-CAG$_{64}$* at twice the levels of *ATXN2-CAG$_{22}$* (2x *ATXN2-CAG$_{22}$*) were co-expressed in the fly eye with the *gmr-GAL4* driver. The three transgenes combined gave expression levels that were 6x *ATXN2-CAG$_{22}$* and compared to the normal control (ctrl), caused degeneration of the external eye (white hatched line, upper panel) and internal retina (double headed arrow, lower panel) that was comparable to the 6x *ATXN2-CAG$_{64}$* (#HZ96R) transgene. Control (ctrl) is *w; UAS-mCD8-GFP/+; gmr-GAL4/+*

B: Quantification of retinal width revealed that co-expression of the three independent 2x *ATXN2-CAG$_{22}$* transgenes caused significant retinal degeneration compared to the normal control (ctrl). The degeneration caused the three independent 2x *ATXN2-CAG$_{22}$* transgenes was similar to the degeneration caused by the 6x *ATXN2-CAA$_{64}$*. Mean (s.e.m.), one-way ANOVA and Tukey's test. **** $p < 0.0001$, ns not significant. Control (ctrl) is *w; UAS-mCD8-GFP/+; gmr-GAL4/+*.

See Supplementary Table S4 for full genotypes.

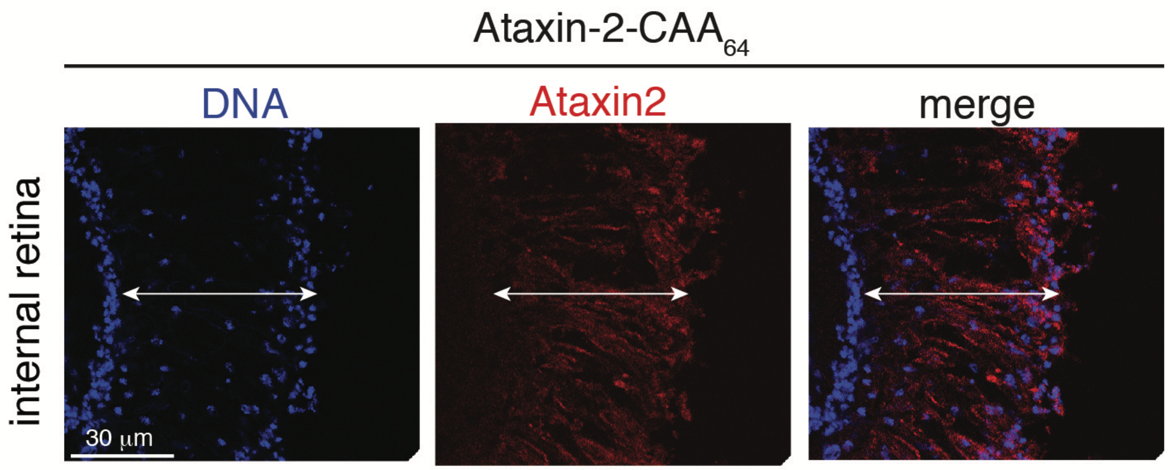

Supplementary Figure S3: Expression of *ATXN2-CAA₆₄* in the *Drosophila* eye leads to a diffuse localization pattern.

Expression of 6x *ATXN2-CAA*$_{64}$ in the eye with the *gmr-GAL4* driver leads to diffuse protein localization in the retina. Shown is a close up image of the internal retinal stained with Hoescht for cell nuclei (blue) and with an antibody to ataxin-2 (red). White arrow indicates retinal depth. See Supplementary Table S4 for full genotypes.

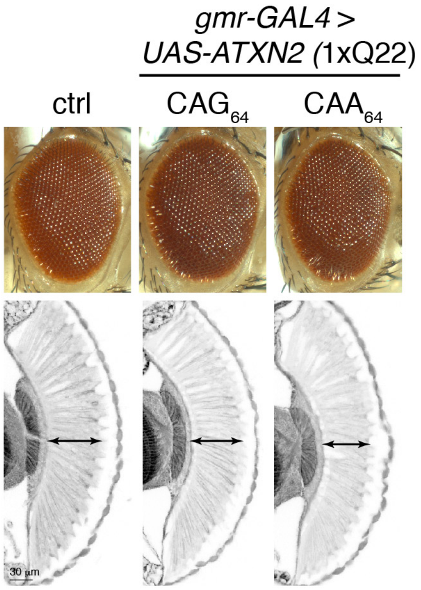

Figure S4: 1x expression of Ataxin-2 with a long CAG or a CAA repeat is not toxic to the eye.

Expression of either *ATXN2-CAG*$_{64}$ or *ATXN-CAA*$_{64}$ in the eye with *gmr-GAL4* confers no observable toxicity to the external (upper panel) or internal (lower panel) eye. Double-headed arrows indicate retinal width. Control (ctrl) is *w; mCD8-GFP/+; gmr-GAL4/+*.

		Transgenic line	Chromosome	Genotype	Repeat number	mRNA levels relative to *ATXN2-CAG22*	*gmr-GAL4* toxicity in the external eye	*gmr-GAL4* toxicity in the internal eye
ATXN2	*CAG-64*	*HZ110*	*2*	*UAS-ATXN2-CAG64 (HZ110)/ SM6a, CyO ; +/+*	64	1	NE	NE
		HZ99	*3*	*+/+ ; UAS-ATXN2-CAG-64 (HZ99)/ TM6c, Sb*	64	2	NE	NE
		HZ100	*3*	*+/+ ; UAS-ATXN2-CAG-64 (HZ100)/TM6c, Sb*	64	2	NE	NE
		HZ106	*2*	*UAS ATXN2-CAG-64 (HZ106)/SM6, CyO ; +/+*	64	2	NE	NE
		HZ96R	*3*	*+/+ ; UAS-ATXN2-CAG-64 (HZ96R)/ TM6c, Sb*	64	6	ModE	ModE
	CAA/G-65	*Mc4202*	*2*	*UAS-ATXN2-CAA/G-65-6M/SM6a, Cy; +/+*	65	2	NE	NE
		Mc4199	*2*	*UAS-ATXN2-CAA/G-65-3M/SM6a, Cy; +/+*	65	3	NE	NE
		Mc4200	*2*	*UAS-ATXN2-CAA/G-65-4M/SM6a, Cy; +/+*	65	6	NE	NE
	CAA-64	*CAA2*	2	*UAS-ATXN2-CAA-64-2M/SM6a, Cy; +/+*	60	1	NE	NE
		CAA5	2	*UAS-ATXN2-CAA-64-5M/SM6a, Cy; +/+*	64	2	NE	NE
		CAA9	3	*+/+; UAS-ATXN2-CAA-64-11M/TM6c, Sb*	63	3	NE	NE
		CAA11	2	*UAS-ATXN2-CAA-64-11M/SM6a, Cy; +/+*	63	6	NE	NE

Key for matched expression group

1
2
3
6

Key for eye toxicity
NE: no effect
ModE: moderate effect

Supplementary Table S1: Transgenic fly lines matched to *ATXN2-CAG$_{22}$* mRNA expression levels

Fly lines were outcrossed to *daughterless gene-switch GAL4* (*daGS-GAL4*) and maintained on RU486 for 48 hr, then RNA was isolated from head and thorax tissue for real-time PCR to determine expression level of the transgenes.

	Parents	Progeny Genotype	Progeny number	
	+/+ ; Daughterless (da)-GAL4 crossed to:		Repeat 1	Repeat 2
Ctrl	*UAS-mCD8-GFP; +/+*	*UAS-mCD8-GFP /+; da-GAL4 /+*	283	379
(1x) Q22	*UAS-ATXN2-Q22; +/+*	*UAS-ATXN2-Q22/+; da-GAL4 /+*	0	0
(1x) Q32	*UAS-ATXN2-Q32/SM6a, Cy; +/+*	*UAS-ATXN2-Q32/+; da-GAL4 /+*	0	0
		+/SM6a, Cy; da-GAL4/+	242	256
(1x) CAA$_{64}^{CAA2}$	*UAS-ATXN2-CAA*$_{64}^{CAA2}$*/SM6a, Cy; +/+*	*UAS-ATXN2- CAA*$_{64}^{CAA2}$*/+; da-GAL4 /+*	0	0
		+/SM6a, Cy; da-GAL4/+	342	274
(2x) CAA$_{65}^{Mc4202}$	*UAS-ATXN2-CAA*$_{65}^{Mc4202}$*/SM6a, Cy; +/+*	*UAS-ATXN2- CAA*$_{65}^{Mc4202}$*/+; da-GAL4 /+*	0	0
		+/SM6a, Cy; da-GAL4/+	254	263
(6x) CAG$_{64}^{HZ96R}$	*+/+ ; UAS-ATXN2-CAG*$_{64}^{HZ96R}$ *TM6c, Sb*	*+/+ ; UAS-ATXN2-CAG*$_{64}^{HZ96R/}$ *da-GAL4*	0	0
		+/+; da-GAL4/TM3, Sb	191	227
(6x) CAA/G$_{65}^{Mc4200}$	*UAS-ATXN2-CAG*$_{65}^{Mc4200}$*/SM6a, Cy; +/+*	*UAS-ATXN2- CAA*$_{65}^{Mc4202}$*/+; da-GAL4 /+*	0	0
		+/SM6a, Cy; da-GAL4/+	207	238
(6x) CAA$_{64}^{CAA11}$	*UAS-ATXN2-CAG*$_{64}^{CAA11}$*; +/+*	*UAS-ATXN2-CAG*$_{64}^{CAA11}$*/+; da-GAL4 /+*	0	0

Supplementary Table S2: Ubiquitous expression of the *ATXN2* transgenes with *da-GAL4* is toxic regardless of the level of transgene expression.

Supplementary Table S3: Reagents

**Fly strains**	**Source**
HZ110: UAS-ATXN2-(CAG)64/SM6a , SM6a, CyO ; +/+	This study
HZ99: +/+ ; UAS-ATXN2-(CAG)64/TM3/ TM3, Sb	This study
HZ100: +/+ ; UAS-ATXN2-(CAG)64/TM3 /TM3, Sb	This study
HZ106: UAS ATXN2-(CAG)64/SM6, CyO ; +/+	This study
HZ96R: +/+ ; UAS-ATXN2-(CAG)64/TM3/ TM3, Sb	This study
Mc4199: UAS-ATXN2-CAA/G-65-3M/SM6a, Cy; +/+	This study
Mc4200: +/+ ; UAS-ATXN2-(CAA/G)65-4M/SM6a, Cy ; +/+	This study
Mc4202: +/+ ; UAS-ATXN2-(CAA/G)65-6M/SM6a, Cy ; +/+	This study
CAA2: UAS-ATXN2-(CAA)64-2M/SM6a, Cy ; +/+	This study
CAA5:UAS-ATXN2-(CAA)64-5M/SM6a, Cy ; +/+	This study
CAA9: +/+; UAS-ATXN2-CAA-64-11M/TM6c, Sb	This study
CAA11: UAS-ATXN2-(CAA)64-11M/SM6a, Cy ; +/+	This study
UAS-ATXN2-CAG-22	(S1)
USA-ATXN2-CAG-32	(S1)
UAS-TDP-43 (37M)/CyO; gmr-GAL4(YH3)/TM6B	(S2)
y^1 w; UAS-mCD8-GFP II*	Bloomington *Drosophila* Stock Center
w^{1118} (also referred to as BL5905)	Bloomington *Drosophila* Stock Center
gmr-GAL4(III)	Y. Hiromi
Elav3A-Gal4	(S3)
Daughterless-Gal4	Bloomington *Drosophila* Stock Center
Daughterless-GeneSwitch-GAL4	(S4)
Spt4.IR: *y[1]sc[*]v[1]sev[21];P{y[+t7.7] v[+t1.8]=TRiP.HMS00685}attP2*	Bloomington *Drosophila* Stock Center
Paf1.IR: *w[1118]; P{GD9782}v20876*	Vienna *Drosophila* Resource Center
eIF4B.IR: *y[1]sc[*]v[1]sev[21];P{y[+t7.7]v[+t1.8]=TRiP.HMS04503}attP40*	Bloomington *Drosophila* Stock Center
eIFH1.IR: *y[1]sc[*]v[1]sev[21]; P{y[+t7.7]v[+t1.8]=TRiP.HMS04504}attP40*	Bloomington *Drosophila* Stock Center
**Antibodies**	
mouse anti-ATXN-2	BD Biosciences, 6113378
mouse anti-Lamin C	DSHB, LC28.26-s
goat anti-mouse HRP	Abcam, ab6789
Rabbit anti-ATXN2	Sigma-Aldrich, HPA 018295
Alexa goat anti-mouse AF568	Invitrogen, A11036
Alexa goat anti-rabbit AF568	Invitrogen, A11011
**ATXN2 primers**	
65caag-1: 5’-CTG TTG CTG CTG CTG CTG TTG CTG CTG CTG CTG CTG CTG CTG CTG TTG CTG CTG CTG CTG TTG CTG CTG CTG CTG TTG CTG CTG CTG CTG CTG CTG CTG CTG CTG GGGCTT CAGCGACATGGTGA GGGGCCC-3’	This study
65caag-2: 5’-CAG CAG CAG CAA CAG CAG CAG CAG CAG CAG CAG CAG CAA CAG CAG CAG CAG CAA CAG CAG CAG CAG CAA CAG CAG CAG CAG CAG CAG CAG CAG CCGC CGC CCGCGG C TGCCAATGTC CGCAAGCCCG-3’	This study
NB1781 5’-GGCAGATCTCCGATGCGCTCAGCGGC-3’	This study
NB1792 5’-AAGAGAGTTGGGACCTGACTGGTA-3’	This study

CAA64-2S: 5'-CAACAA CAACAA CAACAA CAACAA CAACAA CAACAA CAACAA CAACAA CAACAA CAACAA CAACAA CAACAA CAACAA CAACAA CAACAA CAACAA CCGC CGCCCGCGGC TGCCAAT-3'	This study
Sca2-S2: 5'-CGCCGCGTTCCGGCGTCTCC-3'	This study
Sca2-B: 5'-CGGGCTTGCGGACATTGG-3'	This study
**Realtime primers**	
SV40 FP2: 5' TGTGGTGTGACATAATTGGACA 3'	This study
SV40 RP2: 5' TGCTCCCATTCATCAGTTCC 3'	This study
β-Tubulin FP: 5′CATCCAAGCTGGTCAGTG 3'	This study
β-Tubulin RP: 5′ GCCATGCTCATCGGAGAT 3'	This study

Supplementary Table S4: Full genotypes.

**Figure 2**	
ATXN2-CAG$_{22}$	*w;DaGS/UAS-ATXN2-Q22; +/+*
CAG$_{64}$ (HZ110)	*w;DaGS/UAS-ATXN2-CAG*$_{64}$ *(HZ110); +/+*
CAA$_{64}$ (CAA2)	*w;DaGS/UAS-ATXN2-CAA*$_{64}$ *(CAA2); +/+*
CAG$_{64}$ (HZ99)/+	*w;DaGS /+; UAS-ATXN2-CAG*$_{64}$ *(HZ99)/+*
CAA/G$_{65}$ (Mc4202)	*w;DaGS/UAS-ATXN2-CAA/G*$_{65}$ *(Mc4202); +/+*
CAA$_{64}$ (CAA5)	*w;DaGS/UAS-ATXN2-CAA*$_{64}$ *(CAA5); +/+*
CAG$_{64}$ (HZ96R)	*w;DaGS /+; UAS-ATXN2-CAG*$_{64}$ *(HZ96R)/+*
CAA/G$_{65}$ (Mc4200)	*w;DaGS/UAS-ATXN2- CAA/G*$_{65}$ *(Mc4200); +/+*
CAA$_{64}$ (CAA11)	*w;DaGS/UAS-ATXN2-CAA*$_{64}$ *(CAA11); +/+*
**Figure 3**	
ctrl	*w;mCD8-GFP/+; gmr-GAL4/+*
1x lines	
Q22	*w;UAS-ATXN2 Q22/+; gmr-GAL4/+*
CAG$_{64}$	*w;UAS-ATXN2-CAG*$_{64}$ *(HZ110)/+; gmr-GAL4/+*
CAA$_{64}$	*w;UAS-ATXN2-CAA*$_{64}$ *(CAA2)/+; gmr-GAL4/+*
2x lines	
CAG$_{64}$	*w;+ /+; UAS-ATXN2-CAG*$_{64}$ *(HZ99)/ gmr-GAL4*
CAA/G$_{65}$	*w;UAS-ATXN2-CAA/G*$_{65}$ *(Mc4202)/+; gmr-GAL4/+*
CAA$_{64}$	*w;UAS-ATXN2-CAA*$_{64}$ *(CAA5)/+; gmr-GAL4/+*
6x lines	
CAG$_{64}$	*w;+ /+; UAS-ATXN2-CAG*$_{64}$ *(HZ96R)/ gmr-GAL4*
CAA/G$_{65}$	*w;UAS-ATXN2- CAA/G*$_{65}$ *(Mc4200)/+; gmr-GAL4/+*
CAA$_{64}$	*w;UAS-ATXN2-CAA*$_{64}$ *(CAA11)/+; gmr-GAL4/+*
**Figure 4**	
**4A-B**	
ctrl	*w;mCD8-GFP/+; gmr-GAL4/+,*
CAG$_{64}$	*w;+ /+; UAS-ATXN2-CAG*$_{64}$ *(HZ96R)/ gmr-GAL4,*
CAA/G$_{65}$	*w;UAS-ATXN2- CAA/G*$_{65}$ *(Mc4200)/+; gmr-GAL4/+*
CAA$_{64}$	*w;UAS-ATXN2-CAA*$_{64}$ *(CAA11)/+; gmr-GAL4/+*
**4C-D**	
Ctrl	w^{1118}*; +/+; +/+ (5905)*
ATXN2-CAG$_{22}$	*w;DaGS/UAS-ATXN2-Q22; +/+*
CAG$_{64}$ (HZ96R)	*w;DaGS /+; UAS-ATXN2-CAG*$_{64}$ *(HZ96R)/+*
CAA/G$_{65}$ (Mc4200)	*w;DaGS/UAS-ATXN2- CAA/G*$_{65}$ *(Mc4200); +/+*
CAA$_{64}$ (CAA11)	*w;DaGS/UAS-ATXN2-CAA*$_{64}$ *(CAA11); +/+*
**4E**	
ctrl	*w;mCD8-GFP/+; gmr-GAL4/+*
CAA/G$_{65}$	*w;UAS-ATXN2- CAA/G*$_{65}$ *(Mc4200)/+; gmr-GAL4/+*
CAG$_{64}$	*w;+ /+; UAS-ATXN2-CAG*$_{64}$ *(HZ96R)/ gmr-GAL4*
**Figure 5**	
**5A**	

Ctrl	*w;elav3A-GAL4/+; UAS-mCD8-GFP/+; +/+*
CAG_{64} (HZ96R)	*w;elav3A-GAL4/+; +/+; UAS-ATXN2-CAG_{64} (HZ96R)/+*
CAA_{64} (CAA9)	*w;elav3A-GAL4/+; +/+; UAS-ATXN2-CAA_{64} (CAA9)/+*
CAA_{64} (CAA11)	*w;elav3A-GAL4/+; UAS-ATXN2-CAA_{64} (CAA11)/+; + /+*
CAA/G_{65} (Mc4199)	*w;elav3A-GAL4/+; UAS-ATXN2-CAA/G-65-3M/SM6a, Cy; +/+*
CAA/G_{65} (Mc4200)	*w;elav3A-GAL4/+; UAS-ATXN2-CAA/G_{65} (Mc4200)/+; + /+*
**5B**	
da-GAL4 > Ctrl	*w;UAS-mCD8-GFP/+; da-GAL4/+*
CAG_{64} (HZ96R)	*w;+/+; UAS-ATXN2-CAG_{64} (HZ96R)/ da-GAL4*
CAA/G_{65} (Mc4200)	*w;UAS-ATXN2- CAA/G_{65} (Mc4200)/+; da-GAL4 /+*
CAA_{64} (CAA11)	*w;UAS-ATXN2-CAA_{64} (CAA11)/+; da-GAL4 /*
**Figure 6**	
**6A**	
ctrl	*w;mCD8-GFP/+; gmr-GAL4/+*
gmr > TARDBP + ctrl	*w;mCD8-GFP/+; gmr-GAL4, UAS-TARDBP/+*
gmr > TARDBP + Q22	*w;UAS-ATXN2 Q22/+; gmr-GAL4, UAS-TARDBP/+*
gmr > TARDBP + Q32	*w;UAS-ATXN2 Q32/+; gmr-GAL4, UAS-TARDBP/+*
gmr > TARDBP + UAS-ATXN2-CAG64 (1xQ22)	*w;UAS-ATXN2-CAG_{64} (HZ110)/+; gmr-GAL4, UAS-TARDBP/+*
gmr > TARDBP + UAS-ATXN2-CAA_{64} (1xQ22)	*w;UAS-ATXN2-CAA_{64} (CAA2)/+; gmr-GAL4, UAS-TARDBP/+*
**6B**	
ctrl	*w;mCD8-GFP/+; gmr-GAL4/+*
gmr > UAS-ATXN2-CAG64 (1xQ22	*w;UAS-ATXN2-CAG_{64} (HZ110)/+; gmr-GAL4/+*
gmr > UAS-ATXN2-CAA_{64} (1xQ22)	*w;UAS-ATXN2-CAA_{64} (CAA2)/+; gmr-GAL4/+*
**Figure 7**	
Ctrl (healthy)	*w; gmr-GAL4/+*
gmr-GAL4 > 6x UAS-ATXN2-CAG_{64}	*w;UAS-ATXN2-CAG_{64} (HZ96R) gmr-GAL4*
Control for 6XCAG	*w; UAS-mCD8-GFP/+; UAS-ATXN2-CAG_{64} (HZ96R), gmr-GAL4/+*
Spt4.IR	*w ;; UAS-ATXN2-CAG64(HZ96R), gmr-GAL4/TRiP.HMS00685*
Paf1.IR	*w; P{GD9782}v20876/+; UAS-ATXN2-CAG64(HZ96R), gmr-GAL4/+*
eIF4B.IR	*w; TRiP.HMS04503/+; UAS-ATXN2-CAG64(HZ96R), gmr-GAL4/+*
eIF4H1.IR	*w;TRiP.HMS04504/+; UAS-ATXN2-CAG64(HZ96R), gmr-GAL4/+*
**Figure S1**	
ctrl	*w;UAS-mCD8-GFP/+; gmr-GAL4/+*
6x ATXN2-CAG_{64}	*w;+/+; UAS-ATXN2-CAG_{64} (HZ96R)/ gmr-GAL4*
3x (2x ATXN2-CAG_{64})	*w;UAS-ATXN2-CAG_{64} (HZ106)/+; UAS-ATXN2-CAG_{64} (HZ100), UAS-ATXN2-CAG_{64} (HZ99)/gmr-GAL4*
**Figure S2**	
CAA_{64}	*w;UAS-ATXN2-CAA_{64} (CAA11)/+; gmr-GAL4/+*

**Figure S3**	
ATXN2-CAG_{22}	*w; DaGS/UAS-ATXN2-Q22; +/+*
CAG_{64} (HZ110)	*w; DaGS/UAS-ATXN2-CAG_{64} (HZ110); +/+*
CAG_{64} (HZ106)	*w; DaGS/UAS-ATXN2-CAG_{64} (HZ106); +/+*
CAG_{64} (HZ99)	*w; DaGS/+; UAS-ATXN2-CAG_{64} (HZ99)/+*
CAA_{64} (CAA9)	*w; DaGS/+; UAS-ATXN2-CAG_{64} (CAA9)/+*
CAA/G_{64} (Mc4199)	*w; DaGS/UAS-ATXN2-CAG_{64} (Mc4199); +/+*

REFERENCES FOR SUPPLEMENTARY MATERIAL

S1. Kim, H.-J., Raphael, A. R., LaDow, E. S., McGurk, L., Weber, R. A., Trojanowski, J. Q., Lee, V. M.-Y., Finkbeiner, S., Gitler, A. D. and Bonini, N. M. (2014) Therapeutic modulation of eIF2α phosphorylation rescues TDP-43 toxicity in amyotrophic lateral sclerosis disease models. *Nat. Genet.*, **46**, 152–160.

S2. Elden, A. C., Kim, H.-J., Hart, M. P., Chen-Plotkin, A. S., Johnson, B. S., Fang, X., Armakola, M., Geser, F., Greene, R., Lu, M. M., et al. (2010) Ataxin-2 intermediate-length polyglutamine expansions are associated with increased risk for ALS. *Nature*, **466**, 1069–1075.

S3. Hekmat-Scafe, D. S., Dang, K. N. and Tanouye, M. A. (2005) Seizure suppression by gain-of-function escargot mutations. *Genetics*, **169**, 1477–1493.

S4. Tricoire, H., Battisti, V., Trannoy, S., Lasbleiz, C., Pret, A.-M. and Monnier, V. (2009) The steroid hormone receptor EcR finely modulates Drosophila lifespan during adulthood in a sex-specific manner. *Mech. Ageing Dev.*, **130**, 547–552.
